# An Improved Cuckoo Search Optimization Algorithm for the Problem of Chaotic Systems Parameter Estimation

**DOI:** 10.1155/2016/2959370

**Published:** 2016-01-12

**Authors:** Jun Wang, Bihua Zhou, Shudao Zhou

**Affiliations:** ^1^National Key Laboratory on Electromagnetic Environmental Effects and Electro-Optical Engineering, PLA University of Science and Technology, Nanjing 210007, China; ^2^College of Meteorology and Oceanography, PLA University of Science and Technology, Nanjing 211101, China

## Abstract

This paper proposes an improved cuckoo search (ICS) algorithm to establish the parameters of chaotic systems. In order to improve the optimization capability of the basic cuckoo search (CS) algorithm, the orthogonal design and simulated annealing operation are incorporated in the CS algorithm to enhance the exploitation search ability. Then the proposed algorithm is used to establish parameters of the Lorenz chaotic system and Chen chaotic system under the noiseless and noise condition, respectively. The numerical results demonstrate that the algorithm can estimate parameters with high accuracy and reliability. Finally, the results are compared with the CS algorithm, genetic algorithm, and particle swarm optimization algorithm, and the compared results demonstrate the method is energy-efficient and superior.

## 1. Introduction

Chaos is a universal complex dynamical phenomenon, lurking in many nonlinear systems, such as communication systems and meteorological systems. The control and synchronization of chaos has been widely studied [1–4]. Parameter estimation is a prerequisite to accomplish the control and synchronization of chaos. During recent years many parameter estimation methods have been proposed, such as particle swarm optimization (PSO) [5–8], genetic algorithm (GA) [9–12], and mathematical methods of multiple shooting [13]. However, the GA and PSO algorithms are easily trapped into local-best solution that affects the quality of solutions; the precisions of PSO, GA, and multiple shooting are not high enough. Recently, a novel and robust metaheuristic based method called cuckoo search algorithm was proposed by Yang and Deb [14–16]. The algorithm proved to be very promising and could outperform existing algorithms such as GA and PSO [14]. However, the relatively poor ability of local searching is a drawback, and it is necessary to further improve the performance of CS algorithm to obtain a higher-quality solution. The basic principle of the ICS algorithm is to integrate the orthogonal design and simulated annealing operation to enhance the exploitation optimization capacity.

The remaining sections of this paper are organized as follows. In Section 2, a brief formulation of chaotic system parameters estimation is described.  Section 3 elaborates the ICS algorithm, and the results established upon the proposed algorithm and some compared algorithms are given in Section 4. The paper ends with conclusions in Section 5.

## 2. Problem Formulation

A problem of parameter estimation can be converted into a problem of multidimensional optimization by constructing the proper fitness function.

Let the following equation be a continuous nonlinear *n*-dimension chaotic system:

(1) $$\begin{array}{c} \dot{X}=F\left(\;X,X_{0},\theta \;\right), \end{array}$$

where  *X* = (*x*_1_, *x*_2_,…, *x*_*n*_)^*T*^ ∈ *R* ^*n*^ denotes the state vector of the chaotic system, $\dot{X}$ is the derivative of *X*, *X*_0_ = (*x*_10_, *x*_20_,…, *x*_*n*__0_)^*T*^ ∈ *R* ^*n*^ denotes the initial state of system, and *θ* = (*θ*_1_, *θ*_2_,…, *θ*_*d*_)^*T*^ is a set of original parameters. Suppose the structure of the system (1) is known; then the estimated system can be written as

(2) $$\begin{array}{c} \dot{\tilde{X}}=F\left(\;\tilde{X},X_{0},\tilde{\theta }\;\right), \end{array}$$

where $\tilde{X}={\left({\tilde{x}}_{1},{\tilde{x}}_{2},\ldots ,{\tilde{x}}_{n}\right)}^{T}\in R^{n}$ denotes the state vector of the estimated system; $\tilde{\theta }={\left({\tilde{\theta }}_{1},{\tilde{\theta }}_{2},\ldots ,{\tilde{\theta }}_{d}\right)}^{T}$ is a set of estimated parameters. In order to convert the parameter estimation problem into optimization problem, the following objective fitness function is defined:

(3) $$\begin{array}{c} F\left(\;\tilde{\theta }\;\right)=\sqrt{\frac{1}{M}\overset{M}{\underset{i=1}{\sum }}{\left(X-\tilde{X}\right)}^{2}}, \end{array}$$

where *i* = 1,2,…, *M* is the sampling time point and *M* denotes the length of data used for parameter estimation. The parameter estimation of system (1) can be achieved by searching the most proper values of $\tilde{\theta }$ such that the objective function (3) is globally minimized.

It can be found that (3) is a multidimensional nonlinear function with multiple local search optima; it is easily trapped into local optimal solution and the computation amount is great, so it is not easy to search the globally optimal solution effectively and accurately using traditional general methods. In the paper an improved CS algorithm is proposed to solve the complex optimization problem.

## 3. Improved CS Algorithm

### 3.1. Basic CS Algorithm

The basic CS algorithm is based on the brood parasitism of some cuckoo species by laying their eggs in the nests of other host birds. For simplicity in describing the basic CS, the following three ideal rules are used [14]: (1) Each cuckoo lays one egg at a time, and dumps it in a randomly chosen set; (2) the best nests with high-quality eggs will be carried over to the next generations; (3) the number of available host nests is fixed, and the egg laid by a cuckoo is discovered by the host bird with a probability *p*_*a*_ ∈ [0,1]. In this case, the host bird can either get rid of the egg away or simply abandon the nest and build a complex new nest. Based on the above rules, the basic CS algorithm is described as shown in Algorithm 1 [14].

Furthermore, the algorithm used a balanced combination of a local random walk and the global explorative random walk, controlled by a switching parameter *p*_*a*_. The local random walk can be written as

(4) $$\begin{array}{c} x_{i}^{t+1}=x_{i}^{t}+\alpha s⊗H\left(\;p_{a}-\varepsilon \;\right)⊗\left(\;x_{j}^{t}-x_{k}^{t}\;\right), \end{array}$$

where *x*_*j*_^*t*^ and *x*_*k*_^*t*^ are two different solutions selected randomly by random permutation, *H* is a Heaviside function, *ε* is a random number drawn from a uniform distribution, and *s* is the step size.

On the other hand, the global random walk is carried out by using Lévy flights [14–17]:

(5) $$\begin{array}{c} x_{i}^{t+1}=x_{i}^{t}+\alpha ⊕\text{Lévy}\left(\;s,\lambda \;\right). \end{array}$$

Here, *α* > 0 is the step size scaling factor; Lévy(*s*, *λ*) is the step-lengths that are distributed according to the following probability distribution shown in (6) which has an infinite variance with an infinite mean:

(6) $$\begin{array}{c} \text{Lévy}\left(\;s,\lambda \;\right)=\frac{\lambda \Gamma \left(\lambda \right)\sin\left(\pi \lambda /2\right)}{\pi }\frac{1}{s^{1+\lambda }}. \end{array}$$

### 3.2. ICS Algorithm

In order to further improve searching ability of the algorithm, the orthogonal design and simulated annealing operation are integrated into the CS algorithm. The basic idea of the orthogonal design is to utilize the properties of the fractional experiment to efficiently determine the best combination of levels [17]. An orthogonal array of *K* factors with *Q* levels and *M* combinations is denoted as *L*_*M*_(*Q* ^*K*^), where *Q* is the prime number, *M* = *Q* ^*J*^, and *J* is a positive integer satisfying *K* = (*Q* ^*J*^ − 1)/(*Q* − 1). The brief procedure of constructing the orthogonal array *L*_*M*_(*Q* ^*K*^) = [*a*_*i*,*j*_]_*M*×*K*_ is described as shown in Procedure 1.

**Procedure 1: Procedure constructing the orthogonal array.**
*Step  1*. Construct the basic columns
 For *k* = 1 to *J*
  
$j=\frac{Q^{k-1}-1}{Q-1}+1$
  For *i* = 1 to *Q* ^*J*^
   
$a_{i,j}=\left|\frac{i-1}{Q^{J-K}}\right|$mod⁡*Q*
  End for
 End for
*Step  2*. Construct the non-basic columns
 For *k* = 2 to *J*
  
$j=\frac{Q^{k-1}-1}{Q-1}+1$
  For *s* = 1 to *j* − 1
   For *t* = 1 to *Q* − 1
 
*a*_*j*+(*s*−1)(*Q*−1)+1_ = (*a*_*s*_ × *t* + *a*_*j*_)mod⁡*Q*
   End for
  End for
 End for
*Step  3*. Increment *a*_*i*,*j*_ by one for 1 ≤ *i* ≤ *M*, 1 ≤ *j* ≤ *N*

The procedure of the orthogonal design algorithm is elaborated as shown in Algorithm 2 and for more detailed information on the orthogonal design strategy, please refer to [17–19].

The procedure of simulated annealing algorithm is simply stated as shown in Algorithm 3 [20], and for more detailed information on the simulated annealing, please refer to [20–22].

Based on the above description of the orthogonal design strategy and simulated annealing operation, the detailed procedures for parameter estimation with the ICS algorithm can be summarized as shown in Algorithm 4.

## 4. Simulation Results

To demonstrate the effectiveness of the improved algorithm, the algorithm is used to estimate parameters of Lorenz chaotic system [23] and Chen chaotic system [24].

### 4.1. Lorenz Chaotic System

Lorenz chaotic system equation [23] is expressed as follows:

(7) x˙=σ1y−x,y˙=σ2x−xz−y,z˙=xy−σ3z,

where (*x*, *y*, *z*) is the state variables; *σ*_1_, *σ*_2_, *σ*_3_ are the unknown chaotic system parameters which need to be estimated. The real parameters of the system are *σ*_1_ = 10, *σ*_2_ = 28, and *σ*_3_ = 8/3 which ensure a chaotic behavior, in order to obtain the values of some state variables, the fourth-order Runge-Kutta algorithm is used to solve (7), and the integral step is *h* = 0.01. Then a series of state variables values are obtained and 100 state variables of different times ({(*x*(*n*), *y*(*n*), *z*(*n*)),   *n* = 1, 2, …, 100}) are chosen to be the sample data. The parameters of the algorithm are set as follows: the max iteration number is *N* = 200, the sample size is *M* = 100, the annealing mode is shown in (8) where *n* is the iteration number, and the initial temperature is *T*_0_ = 100. Consider

(8) $$\begin{array}{c} T\left(\;n\;\right)=\frac{T_{0}}{\ln\left(1+n\right)}. \end{array}$$

The objective (fitness) function *H* is shown in (9), where (*x*(*n*), *y*(*n*), *z*(*n*)) is the *n*th state variable that corresponds to the true system parameters and$\;(\tilde{x}(n),\tilde{y}(n),\tilde{z}(n))$ is the *n*th state variable that corresponds to the estimated system parameters:

(9) H=1M∑n=1Mx~n−xn2+y~n−yn2+z~n−zn2.

**Figure 1:**
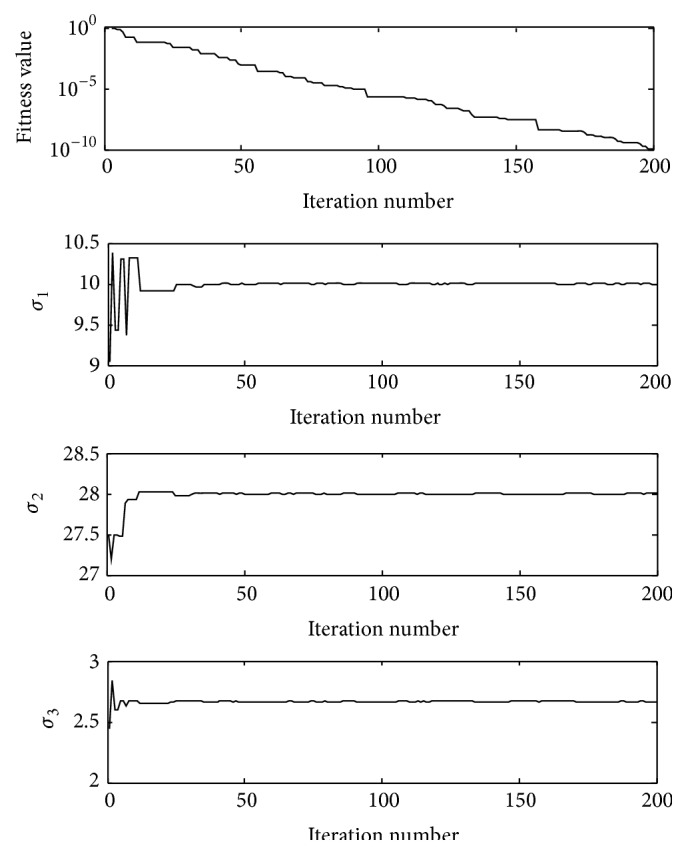
The convergence process of fitness function value and three parameters (*σ*_1_, *σ*_2_, *σ*_3_) during the iterations in a single experiment under the noiseless condition.

Figure 1 shows the convergence process of the fitness values and three parameters (*σ*_1_, *σ*_2_, *σ*_3_) during the iterations in a single experiment.

In order to eliminate the difference of each experiment, the algorithm is also executed 50 times; then the mean value of the 50 experiments is taken as the final estimated value; the mean value and best value of the 50 experiments are listed in Table 1. The results based on CS (the best parameter setting is *p*_*a*_ = 0.25, *p*_*a*_ = 0.01), PSO (the best parameter setting is *w* = 0.8, *c* = 1.5, where *w* is the inertia weight and *c* is acceleration factor), and GA (the best parameter setting is *c*_*r*_ = 0.8, *m*_*u*_ = 0.1, where *c*_*r*_ is the crossover rate and *m*_*u*_ is the mutation rate) are also listed in Table 1.

It can be seen from Table 1 that the best fitness values obtained by ICS algorithm are quite better than the other algorithms. The mean values of the established parameters are also with higher precision than others. The estimated values are close to the true values infinitely. It can be concluded in general that the ICS algorithm contributes to superior performance, CS performs nest-best, PSO is better than GA, and GA performs worst.

**Figure 2:**
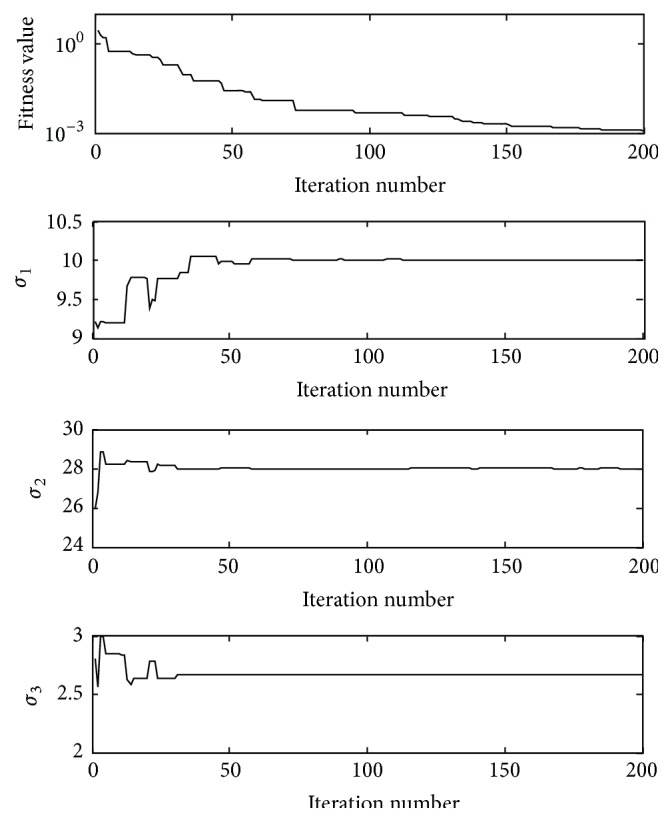
The convergence process of fitness function value and three parameters (*σ*_1_, *σ*_2_, *σ*_3_) during the iterations in a single experiment under the noise condition.

As the actual chaotic systems always associate with noise, in order to test the performance of parameter estimation in the noise condition, the noise sequences are added to the original sample data. The white noise is added to the state variables {(*x*(*n*), *y*(*n*), *z*(*n*)),   *n* = 1, 2, …, 100}; the range of the noise sequences is from −0.1 to 0.1.  Figure 2 shows the convergence process of the fitness values and three parameters (*σ*_1_, *σ*_2_, *σ*_3_) during the iterations in a single experiment under the noise condition.

In order to eliminate the difference of each experiment, the algorithm is executed 50 times, then the mean value of the 50 experiments is taken as the final estimated value, and the corresponding results are listed in Table 2.

It can be seen from Table 2 that the four algorithms all have a certain capability of identification of parameters, but the performance of ICS is much better than the other algorithms; it supplies more robust and precise results; although the precision of the estimated parameters is declined compared with the results in the noiseless condition, the precision is still satisfactory. Then it can be concluded that the ICS algorithm possesses a powerful capability for parameters identification in the noise condition.

### 4.2. Chen Chaotic System

**Figure 3:**
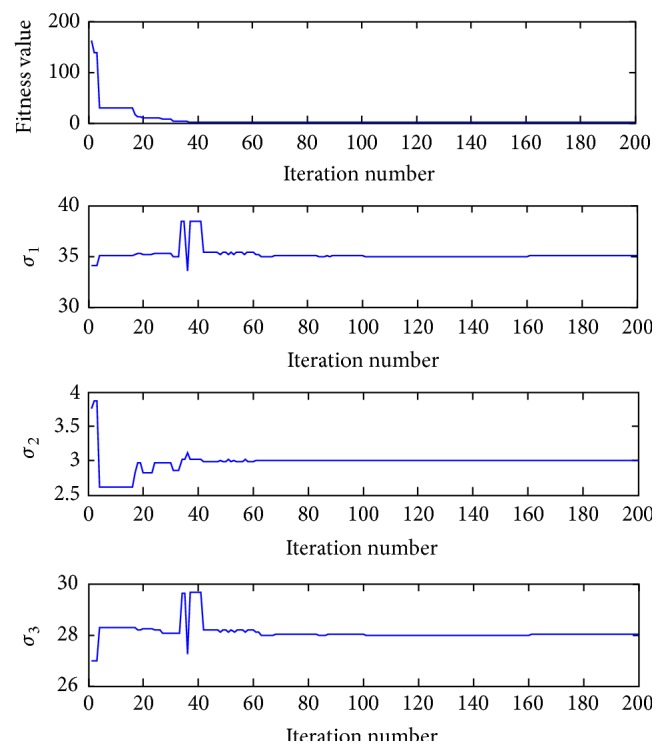
The convergence process of fitness function value and three parameters (*σ*_1_, *σ*_2_, *σ*_3_) during the iterations in a single experiment under the noiseless condition.

Chen chaotic system equation [24] is expressed as follows:

(10) x˙=σ1y−x,y˙=σ3−σ1x−σ3y−xz,z˙=xy−σ2z,

where (*x*, *y*, *z*) is the state variables; *σ*_1_, *σ*_2_, *σ*_3_ are the unknown chaotic system parameters which need to be estimated. The real parameters of the system are *σ*_1_ = 35, *σ*_2_ = 3, and *σ*_3_ = 28 which ensure a chaotic behavior, the fourth-order Runge-Kutta algorithm is used to solve (10), and the integral step is *h* = 0.01. Then a series of state variables values are obtained and 100 state variables of different times ({(*x*(*n*), *y*(*n*), *z*(*n*)),   *n* = 1, 2, …, 100}) are chosen to be the sample data. The parameters of the algorithm are set as follows: the max iteration number is *N* = 200, the sample size is *M* = 100, the annealing mode is shown in (8) where *n* is the iteration number, and the initial temperature is *T*_0_ = 100. The convergence process of the fitness values and three parameters (*σ*_1_, *σ*_2_, *σ*_3_) during the iterations in a single experiment is shown in Figure 3. In order to eliminate the difference of each experiment, the algorithm is executed 50 times, then the mean value of the 50 experiments is taken as the final estimated value, and the corresponding results are listed in Table 2.

It can be seen from Table 3 that the best fitness values obtained by ICS algorithm are quite better than the other algorithms. The mean values of the established parameters are also with higher precision than others. The estimated values are close to the true values asymptotically. It can be concluded in general that the ICS algorithm contributes to superior performance, CS performs nest-best, PSO is better than GA, and GA performs worst.

**Figure 4:**
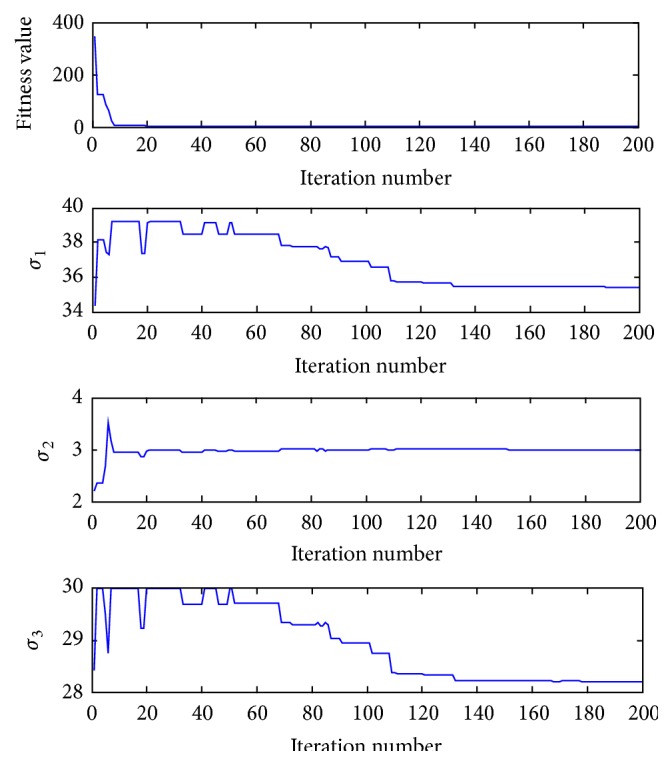
The convergence process of fitness function value and three parameters (*σ*_1_, *σ*_2_, *σ*_3_) during the iterations in a single experiment under the noise condition.

As the actual chaotic systems always come along with noise, in order to test the performance of parameter estimation in the noise condition, the noise sequences are added to the original sample data. The white noise is added to the state variables {(*x*(*n*), *y*(*n*), *z*(*n*)),   *n* = 1, 2, …, 100}; the range of the noise sequences is from −0.1 to 0.1.  Figure 4 shows the convergence process of the fitness values and three parameters (*σ*_1_, *σ*_2_, *σ*_3_) during the iterations in a single experiment under the noise condition.

It can be seen from Table 4 that the four algorithms all have a certain capability of identification of parameters, but the performance of ICS is much better than the other algorithms; it supplies more robust and precise results; although the precision of the estimated parameters is declined compared with the results in the noiseless condition, the precision is still satisfactory. Then it can be concluded that the ICS algorithm possesses a powerful capability for parameters identification in the noise condition.

## 5. Conclusion

In this paper, an energy-efficient and superior ICS algorithm is proposed to estimate chaotic system parameters. The estimated results demonstrate the strong capabilities and effectiveness of the proposed algorithm, compared with the CS, PSO, and GA algorithms; the ICS algorithm supplies more robust and precise results. Besides, the algorithm also has a more powerful capability of noise immunity. In general, the proposed ICS algorithm is a feasible, energy-efficient, and promising method for parameters estimation of chaotic systems.

## Figures and Tables

**Algorithm 1:**
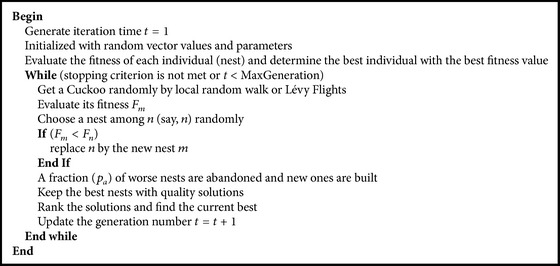
Basic cuckoo search algorithm.

**Algorithm 2:**
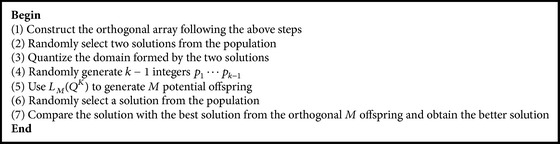
Orthogonal design algorithm.

**Algorithm 3:**
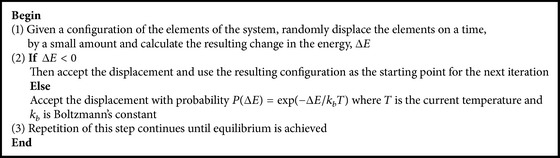
Simulated annealing algorithm.

**Algorithm 4:**
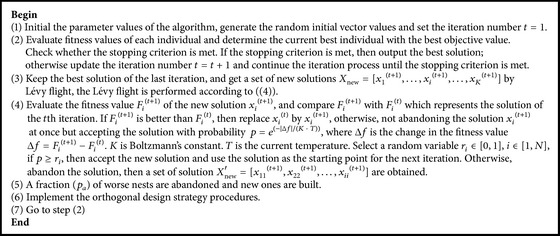
Improved cuckoo search algorithm.

**Table 1:** The statistical results based on different methods in the noiseless condition.

	Mean value				Best value			
	ICS	CS	PSO	GA	ICS	CS	PSO	GA
*σ*_1_	10.000000	9.998736	9.985012	10.082051	10.000000	9.999927	9.995510	10.026911
*σ*_2_	28.000000	28.000005	28.014411	27.881034	28.000000	28.000002	28.001304	28.004702
*σ*_3_	2.666667	2.666661	2.668102	2.681882	2.666667	2.666665	2.666802	2.669018
*H*	1.1822*e* − 010	3.7614*e* − 004	0.069517	0.331901	1.2933*e* − 011	2.9556*e* − 005	0.011377	0.113969

**Table 2:** The statistical results by different algorithms in the noise condition.

	Mean value				Best value			
	ICS	CS	PSO	GA	ICS	CS	PSO	GA
*σ*_1_	9.996110	10.080014	9.844606	10.217998	9.998941	10.001565	9.881002	10.044011
*σ*_2_	28.002272	27.980212	27.860013	27.661201	28.000099	28.001995	28.022441	27.900189
*σ*_3_	2.666590	2.658890	2.700198	2.659880	2.666675	2.667704	2.675596	2.670228
*H*	0.008402	0.0390389	0.221401	0.500227	0.000908	0.001228	0.050931	0.255996

**Table 3:** The statistical results by different algorithms in the noiseless condition.

	Mean value				Best value			
	ICS	CS	PSO	GA	ICS	CS	PSO	GA
*σ*_1_	34.999438	35.089675	34.844278	33.535396	34.999945	34.996661	34.782290	35.102699
*σ*_2_	2.999951	2.999081	3.012977	3.005031	2.999999	2.999907	2.998694	2.991955
*σ*_3_	27.999757	28.043810	27.917648	27.291109	27.999974	27.998427	27.895888	28.053975
*H*	4.6438*e* − 007	4.6628*e* − 004	0.027312	0.115259	2.9658*e* − 010	3.2051*e* − 006	0.003312	0.010562

**Table 4:** The statistical results by different algorithms in the noise condition.

	Mean value				Best value			
	ICS	CS	PSO	GA	ICS	CS	PSO	GA
*σ*_1_	35.421272	35.859540	34.108398	35.962547	34.970096	35.112205	35.313896	10.044011
*σ*_2_	2.996796	2.993281	2.970277	3.052984	2.999311	2.997309	2.980455	27.900189
*σ*_3_	28.204815	28.418425	27.588450	28.434651	27.985940	28.055617	28.162608	2.670228
*H*	0.009334	0.038112	0.244276	0.591957	1.5986*e* − 004	0.001495	0.062901	0.255996
